# Metabolic Activity of Micromycetes Affecting Urban Concrete Constructions

**DOI:** 10.1155/2018/8360287

**Published:** 2018-12-02

**Authors:** Galina Yakovleva, Eugene Sagadeev, Viktor Stroganov, Olga Kozlova, Rodion Okunev, Olga Ilinskaya

**Affiliations:** ^1^Institute of Fundamental Medicine and Biology, Kazan (Volga Region) Federal University, 420008 Kazan, Kremlevskaya Str., 18, Russia; ^2^Kazan State University of Architecture and Engineering, 420043 Kazan, Zelenaya Str., 1, Russia

## Abstract

Concrete resistance to the destructive action of microorganisms is considered as a measure of its durability and is increasingly being raised as an important issue. We focused our study on the biodeterioration of concrete specimens widely used as a building material of urban houses by micromycetes isolated from the inner wall surface of the former military hospital in Kazan city, Tatarstan, Russia. Fungal community consists of 9* Penicillium* isolates, 6* Aspergillus*, 2* Trichoderma*, and 1 isolate of* Alternaria*. First, we have identified two dominant isolates,* Aspergillus fumigatus* and* Penicillium brevicompactum*, and characterized their destructive properties according to the radial growth rate, antagonistic activity towards bacterial habitants of concrete, and production of organic acids. Then, we have demonstrated that five tested brands of high-strength concrete differ in bioreceptivity. The alterations in concrete resistances to compression and flexure after fungal attack were recorded at the trend level, mainly due to a short exposure time of concrete to fungal destructors in tests recommended by national Russian standard. Finally, using scanning electron microscopy we have shown that colonization of concrete by the dominant fungi includes their penetration into the thickness of concrete and germination in cracks. Elementary analysis revealed the decrease of calcium content on about 41% after fungal growth on the concrete in liquid phase and on 32% by superficial growth in comparison with the samples without fungal treatment.

## 1. Introduction

Concrete, a mixture of Portland cement and water, aggregates and in some cases admixtures is a building material that gains strength over time. Biogenic deterioration of the concrete traditionally referred to microorganisms. The causes of building material biodeterioration are three main processes: mechanical, assimilative (building materials are a source of nutrition and energy for microorganisms), and dissimilative (the interaction of building materials with aggressive metabolites of microorganisms) [1]. The action of microorganisms on concrete structures can be classified according to their effects on concrete surfaces, concrete matrices and on cracking and crack growth [2]. The growth effects are supplemented by chemical action of microbial metabolites leading to the alteration of physicochemical properties of concrete materials and accelerating the destruction process [3]. The influence of microorganisms on concrete mainly occurs by eroding a concrete surface. This leads to an increase in the porosity of concrete, the appearance of cracks, chips, and other damage, which ultimately significantly reduces the life of this material [4]. The favorable conditions for microbial growth on the concrete surfaces are increased humidity (i.e., between 60% and 98%), long-term cycles of wetting and drying, freezing and thawing, high concentration of carbon dioxide, high concentration of Cl^−^ or SO_4_^2−^ ions, and a small amount of acids [5]. Biodeterioration of concrete structures was demonstrated to be a common occurrence; however, various microbial populations and mechanisms were shown to play different roles at different locations [6]. We focused our study on the biodeterioration of concrete specimens widely used as a building material of urban houses.

Many buildings are believed to have been damaged to varying degrees by microbiologically induced destruction of concrete. Until today, there is no clear understanding of the extent and severity of the damage, the operative mechanism, and effective remedial techniques for the microbial attack to concrete structures [7]. There is a set of investigations describing concrete biodeterioration in tropical coastal and marine areas [8, 9], Coombes, 2016, but little is known about this process in cities located in temperate-continental climate zones. Biologically influenced corrosion of concrete in big cities has most often been detected in building foundations and walls. Taken into account the absence of direct contacts of the city buildings with groundwater and low winter temperatures, the role of nonsporeforming and sporeforming but thermophilic sulfate-reducing bacteria (genus* Desulfotomaculum*) in destruction of houses is minimal. The basic destructors in this area are micromycetes synthesizing broad spectrum of organic acids which can be extremely aggressive to concrete [10]. Moreover, production of secondary metabolites (antibiotics, mycotoxins) gives them an advantage in the concrete colonization over bacteria.

Our study is aimed at isolation and identification of filamentous fungi growing on the concrete walls of old buildings in Kazan city (Tatarstan, Russia) and characterization of their destructive properties based on the measurement of growth rate and acid production, for the future assessment of deterioration level of fine-grained concrete induced by them. Applying an integrated approach combining methods of microbiology with standard methods for determining the strength of building structures, we justified the connection between the decrease of concrete strength and the level of fungal metabolic activity whose main markers were production of organic acids, radial growth rate, and antagonistic activity.

## 2. Materials and Methods

### 2.1. Micromycetes

The microscopic fungi were isolated from the inner wall surface of the former military hospital in Kazan city (Tatarstan, Russia). The surface of the concrete wall with visible growth of micromycetes at 10 different sites was treated with a sterile cotton swab, which was then placed in a tube with 5 ml of sterile water. From there, 10 samples of 100 ml (in triplicates) were applied to a solid Czapek-Dox medium in Petri dishes, which were incubated for 5 days at 30°C. The morphology of colonies was analyzed microscopically. Total number of isolates was 18. The dominant fungi of the genera* Aspergillus* and* Penicillium* were used to study their destructive properties. For the cultivation of micromycetes, a liquid and agarized Czapek-Dox medium was used (g/l): NaNO_3_ - 2.0; KH_2_PO_4_ - 1.0; MgSO_4_×7 H_2_O-0.5; KCl - 0.5; FeSO_4_ - 0.01; sucrose - 30.0; H_2_O - 1000 ml. Microscopic identification of dominant fungi was supplemented by Sanger sequencing of ITS (internal transcribed spacer) regions of the isolates using standard primers ITS1 and ITS 4 [11]. Variable ITS1 and ITS2 sequences which are located in the ribosomal operon between the small (SSU) and the large subunit (LSU) sequences surrounding the 5.8 S rRNA gene were analyzed and compared with those of reference strains downloaded from MycoBank and GenBank databases.

### 2.2. Concrete Specimens and Their Treatment by Fungi

**Figure 1:**
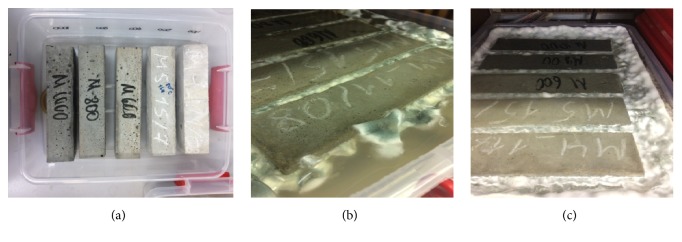
Concrete specimens of different marks (M300, M400, M600, M800, and M1000), untreated, indicating the location of samples before covering by the medium (**a**) and treated by* Aspergillus fumigatus* A2 (**b**) and* Penicillium brevicompactum* P4 (**c**) spores after 5 days of fungal growth. The photos were performed after opening the containers, and the covers were removed. The signs on the beams correspond to the tested brands of concrete.

High strength sandy concrete samples M 400, M 500, М 600, М 800, and М 1000 were made in the form of beams with a size of 160×40×40 mm. In the manufacture of cement-sand mortars based on Portland cement grades PC400 and PC500 (strength corresponding to classes B30 and B40) and concretes of classes B45, B60, and B70 the ratio of sand (fraction 0.5-0.25 mm corresponding to Russian state standard GOST 8736-93) and cement was three to one. In the manufacture of concrete class B45, B60, and B70 (М 600, М 800, and М 1000) based on Portland cement PC500, Melflux 2651 F superplasticizer (BASF Construction Polymers) was added in an amount of 0.5% by weight of cement (Mordovcement, Russia). The water/cement ratio was 53/100. For universality of notation, hereinafter we use the term “cement” for all samples. The strength of concrete is specified by the marks denoted by the Latin letter “M” and by numbers from 400 to 1000, depending on the amount of cement used in a mix and indicating the compressive strength in kg/cm^2^. The prepared sterile beams were placed in containers presterilized with 70% ethanol (Figure 1(a)). Containers were filled with a sterile medium, and spores of dominant micromycetes (10^5^/ml) were added (Figures 1(b) and 1(c)). Control container was filled by sterile Czapek-Dox medium without inoculation. The lower part of the beams up to a height of 2 cm was immersed in the medium; the upper part was in the humid atmosphere of the closed containers which were incubated at 30°C during 28 days.

### 2.3. Organic Acids Analysis

Cultural fluid of 18 fungal isolates growing 5 days up to the end of exponential phase on liquid Czapek-Dox medium was analyzed on the production of secreted organic acids using a high-performance liquid chromatograph Flexar (PerkinElmer, USA). The separation of organic acids was carried out in a Brownlee Analytical C18 (150 × 4.6 mm) column packed with 3 *μ*m particles. The elution was carried out at room temperature in a linear gradient using a system consisting of a solution of 10 mM KH_2_PO_4_ adjusted with orthophosphoric acid to pH 2.4 (eluent A) and acetonitrile (eluent B).The flow rate of the mobile phase was 1 ml/min. Peaks were identified using a UV detector at 210 nm [12]. As standards, acid solutions were used (g/l): oxalic (0.15), tartaric (0.35), malic (0.5), lactic (0.35), citric (0.5), and acetic (0.15).

### 2.4. Determination of Micromycete Radial Growth Rate

The rapid growth rate of fungi is connected with the production of organic acids as primary metabolites. To found the most active fungal isolates, the determination of micromycete radial growth rate (RGR) was performed. The colony diameter of isolates on agar medium was measured each 24 h in eight mutually perpendicular directions; arithmetic mean was taken at each time-point. The RGR was calculated according to the formula: RGR = (r - r_o_)/(t - t_o_), where r_o_ is the radius of the colonies at the initial time t_o_ and r is the radius of the colonies at the time t.

### 2.5. Antagonistic Activity Assay

Secondary metabolites producing at stationary growth phase are not involved directly in the normal growth and development of the fungus, but they play an important role in ecological interactions with other organisms. To characterize the ability of fungi to produce some aggressive secondary metabolites contributing to inhibition of bacterial community growth on concrete and indirectly affect the destruction of concrete, the antagonistic activity of micromycetes towards isolated bacterial community was determined by the agar block method. Flushing water from the concrete surface was added to LB medium and cultivated for 24 hours to obtain the mixed bacterial culture. The micromycete isolates A2 and P4 were cultured for 10 days on the Czapek-Dox agar. Then, agar blocks with growing fungi were cut with a sterile plug drill and transferred to the surface of the Czapec-Dox agar, freshly seeded with a bacterial culture, and incubated for 2 days at 28°C. The antagonistic activity was measured by the size of bacterial growth inhibition zone.

### 2.6. Concrete Stability Assay

Compressive and flexural strengths are measured by breaking concrete specimens in special hydraulic press and are calculated as failure load divided by cross-sectional area (reported in megapascals, MPa). Compressive and flexural strengths were tested before and after exposure to growing mycromycetes as described earlier [13].

### 2.7. Scanning Electron Microscopy

Concrete samples were washed, fixed by glutaraldehyde (if fungal growth was found), dehydrated with ethanol and dried at room temperature. Before microscopy samples were coated with gold/palladium. The determination of the element composition and visualization of concrete surface structure were carried out using scanning electron microscopy (Carl Zeiss Merlin with energy dispersion Spectrometer AZtec X-MAX, Germany). Surface morphology was analyzed at an accelerating voltage of 5 keV. Elemental analysis was carried out at an accelerating voltage of 20 keV and a working interval of 9 mm. A set of Aztec program standards (reference standards for X-RAY microanalysis “Registered Standard No. 8842”) was used.

### 2.8. Statistics

Mathematical processing of data was carried out using standard computer program Excel 7.0. All experiments were carried out in at least three replicates. The data group was considered homogeneous if the root-mean-square deviation *σ* in it did not exceed 12%.

## 3. Results

### 3.1. Analysis of Isolated Mycromycetes and Their Organic Acids

We have isolated 18 micromycetes from the inner walls of the old hospital building in Kazan city and found that fungal communities represented the class* Eurotiomycetes* and family* Trichocomaceae*. Predominant genera (83%) were filamentous fungi* Penicillium* (9 isolates) and* Aspergillus *(6 isolates). Micromycetes of the genera* Alternaria* (1 isolate) and* Trichoderma* (2 isolates) were found to be minor representatives.

**Figure 2:**
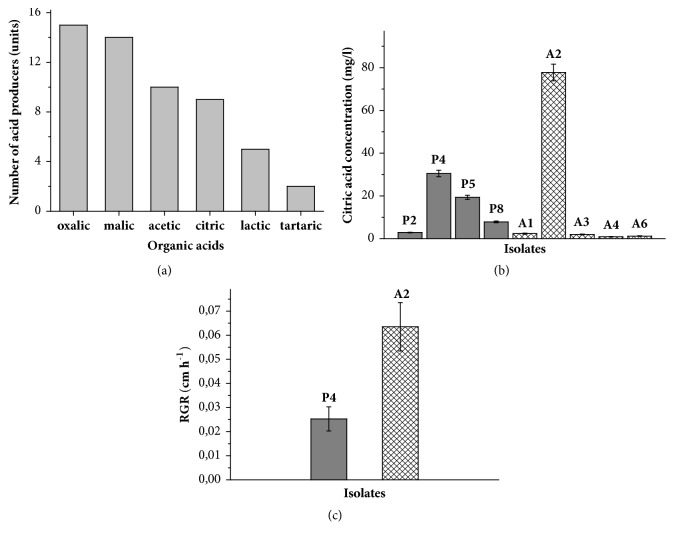
Metabolic activity of micromycetes after 5 days of cultivation. (**a**) The number of isolates synthesizing different organic acids; (**b**) the amount of citric acid produced by* Aspergillus *isolates (marked with letter A) and* Penicillium* isolates (marked with letter P); (**c**) radial growth rate of two dominant isolates,* A. fumigatus* A2 and* P. brevicompactum* P4.

**Figure 3:**
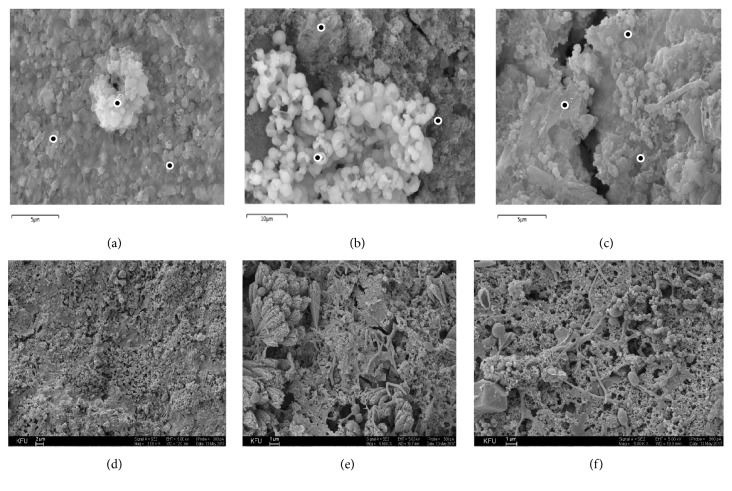
Visualization of M 600 concrete samples surface infected by* P. brevicompactum* P4 spores after 28 days of treatment. (**a, d**) Control samples without treatment; (**b, e**) fungal growth on the concrete surface which was completely immersed in the culture medium; (**c, f**) superficial growth on the concrete surface which was contacted with air in container. (**a-c**) Samples used for element composition testing, points show areas for analysis (Merlin scanning microscope, Carl Zeiss, Germany). (**a**) Illustration of chemical formation of calcite around the microcrack.

Figure 2 demonstrates the metabolic activity of micromycetes after 5 days of cultivation according to the production of organic acids. The 15 investigated isolates, excluding* Alternaria* and* Trichoderma*, are able to synthesize oxalic acid. Malic acid was found in cultural fluid of 14 micromycetes, acetic acid was synthesized by 10 isolates, citric—by 9, lactic—by 5, and tartaric acid—by two isolates (Figure 2(a)). Oxalic acid, when contacted with the concrete surface, forms a complex of calcium oxalate which has a low solubility and therefore contributes to the formation of a protective layer on the concrete surface which promotes the preservation and strengthening of concrete structure, as well as diminishing of microcracks formation [14]. Nonsoluble calcium tartrate could have the same consequences. The acetic, lactic, and malic acids did not form the nonsoluble salts and thus are more aggressive towards concrete structures. Calcium citrate is slightly soluble in water and freely soluble in diluted acids, especially at lower temperatures (so-called “inverse solubility”) [15]. When calcium citrate has shown crystalline precipitate formation, it has been attributed to the salt's superior solubility over that of calcium carbonate formed by contact with carbon dioxide as calcite tightly associated with concrete surface (Figure 3(a)). In this regard, to study the effect of organic acids on concrete stability we selected micromycetes synthesizing the greatest amount of citric acid.

Of the nine isolates capable of synthesizing citric acid four isolates belonged to the genus* Penicillium *(P1, P2, P3, and P4) and five to the genus* Aspergillus* (A1, A2, A3, A4, and A6) (Figure 2(b)). The maximum amount of citric acid among these isolates was produced by* Aspergillus* A2 (77.74 mg/l). Despite the fact that this A2 isolate had the highest concentration of citric acid in the culture liquid, the percentage ratio of citric acid among all other produced organic acids (their joint content was taken for 100%) was only 12.5%. The maximum percentage ratio of citric acid (29.0%, corresponds to 30.47 mg/l) was detected for* Penicillium *P4. Based on this data and the measured values of RGR (Figure 2(c)) we selected two isolates identified as* A. fumigatus* A2 and* P. brevicompactum* P4 for the treatment of concrete samples. ITS region sequences are represented in Supplementary.

### 3.2. Characterization of Micromycete Growth and Antagonistic Activity

**Figure 4:**
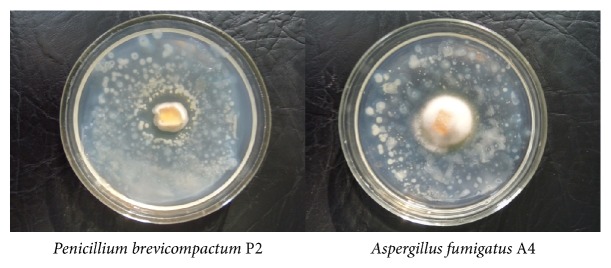
Antagonistic activity of micromycetes towards the bacterial community. The transparent zone around the agar block appearing after 2 days of incubation at 28°C reflects the inhibition of bacterial growth by secreted secondary metabolites of fungi.

Already on the 5^th^ day of the experiment, growth of* A. fumigatus* A2 and* P. brevicompactum* P4 on the all concrete samples was appeared (Figures 1(b) and 1(c)). The growth of* A. fumigatus* A2 was significantly more intensive than the growth of* P. brevicompactum* P4 confirming the belonging of A2 isolate to r-strategists. R-strategists have concurrent characteristics based on the ability to rapidly occupy a new habitat [16]. According to RGR and full spectra of produced acids, the most aggressive were both the strains* A. fumigatus* A2 and the* P. brevicompactum* P4, wherein the strain A2 possessed the highest RGR (Figure 2(c)) and P4 strain had the highest antagonistic activity against bacterial community isolated from the same walls of the old hospital building (Figure 4).

### 3.3. Modification of Concrete Stability and Element Composition

**Figure 5:**
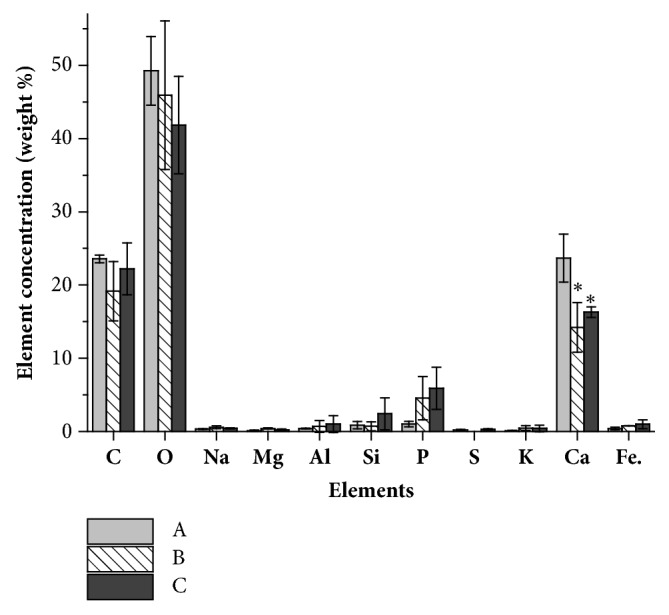
Content of elements in the surface layer of M 600 concrete.** A:** without treatment;** B**: surface which was completely immersed in the culture medium during 28days of* P. brevicompactum* P4 growth;** C**: concrete surface which was contacted with air in container during 28days of P4 growth. Each column represents mean measurement of three points located on the concrete surface shown in Figures 2(a)–2(c). Significant differences from option A (p <0.05) are indicated by *∗*.

Russian state standard (GOST 9.048-89: *«*Unified system of corrosion and ageing protection. Technical items. Methods of laboratory tests for mould resistance*»*) recommended treating the samples by fungi during 28 days. After 28 days of contact with the strain* A. fumigatus* A2 and* P. brevicompactum* P4 in containers partially filled with medium, the values of concrete samples stability were determined. As it can be seen from Table 1, the coefficients of resistance to flexure (R_f_) and to compression (R_c_) after contact with the growing strains decreased by no more than 1% indicating that there are low changes in the concrete specimens strength. The minimal changes were also observed when the samples were incubated in medium without inoculation (Table 1). However, these changes increase over time and gradually contribute to the deterioration of concrete. Penetration of* P. brevicompactum* P4 into the thickness of concrete and germination in cracks was established using scanning microscopy (Figure 3). The same results were obtained for* A. fumigatus* A2 (data not shown). It should be noted that fungal growth on the surface of concretes which were completely immersed to a depth of 5 cm in the culture medium (Figures 3(b) and 3(e)) was more intensive compared to superficial one at the border with air (Figures 3(c) and 3(f)). This fact was confirmed by element analysis of concrete surface samples. As can be seen from Figure 5, the decrease of calcium content on about 41% after fungal growth on the concrete in liquid phase was slightly higher than this decrease (32%) by superficial growth. No significant changes in the content of other elements were found.

## 4. Discussion

### 4.1. Isolates from Buildings Differ from Listed in Recommendations for Biostability Testing

The reason for the concrete destruction of city buildings is mainly the incorrect operating conditions, rather than the spontaneous effects of atmospheric precipitation. The lack of room ventilation, high humidity, a large crowd of people, and, finally, a long time of operation all cause a slow, but permanent destruction of concrete structures used for internal floors of residential buildings. That is why our research is aimed at identifying microorganisms that destroy concrete in the real operating conditions of buildings. It was important to determine how different types of concrete used in construction are susceptible to biodeterioration and how standard tests to determine it reflect the real situation. To date, there are more than 200 different methods to test biostability of building materials. Along with international standards, there are national standards for individual countries (STD 141C/6271/2-86 for USA; BS 1133 for UK, DIN 53739-84 for Germany, NF X 41-514-81 for France; JIS Z 2911-87 for Japan, GOST 9.048-89 for Russia). GOST 9.048-89 recommends the application of 8 different fungi (*Aspergillus niger, Aspergillus terreus, Aureobasidium pullulans, Paecilomyces variotii, Penicillium funiculosum, Penicillium ochrochloron, Scopulariopsis brevicaulis, *and* Trichoderma viride*) to test alteration of concrete strength. According to our data, the species isolated from the old building walls differ from recommended ones. This means that the biodeteriogen microorganisms are special for each particular building which they inhabit. Micromycetes isolated in this study mainly belong to the genera* Aspergillus *and* Penicillium*. The species identification was carried out for two more aggressive isolates,* A. fumigatus* A2 and* P. brevicompactum* P4, which were selected for analysis of their biodeteriogen activity towards concrete samples because of high level of primary metabolites synthesis (the amount of citric acid produced by* A. fumigatus* A2 was 77.74 mg/l, and by* P. brevicompactum* P4 was 30.47 mg/l), as well as because of high growth rate which provides the advantage in settling ecological niches compared to slow-growing microorganisms.

The main fungal genera detected on painted buildings over the 42-week period were* Alternaria, Curvularia, Epicoccum, Helminthosporium, Coelomycetes* (mainly* Pestalotia/Pestalotiopsis*),* Monascus, Nigrospora, Aureobasidium*, and* Cladosporium* [17]. A list of major strains in hospital environments (Centre F. Baclesse, Normandy, France) compiled according to their frequency, concentration level, and/or capacity to produce mycotoxins in vitro was as follows:* A. fumigatus, A. melleus, A. niger, A. versicolor, Cladosporium herbarum, Purpureocillium lilacinum, and P.brevicompactum* [18]. At this list there are two representatives (*A. fumigatus and P. brevicompactum*) which we have also isolated from the walls of old hospital in Kazan. So, it could be concluded that biodeteriogen fungi isolated from concrete walls of buildings completely differ from isolates from painted surfaces.

### 4.2. Organic Acids Spectra Play an Important Role in Concrete Biodeterioration

Adverse effects of geoactive fungi include biodeterioration of natural and synthetic materials, rock and mineral-based building materials (e.g., concrete), cultural heritage, metals, alloys, and related substances [19]. There are three key biological factors affecting the destruction of concrete in a certain building: the presence of fungal strains having a high growth rate and as a consequence dominating in the community (i); high level of produced organic acids, especially citric acid, whose salts with calcium do not form a protective film but provoke microcracks deepening (ii); significant antagonistic activity of certain fungi strains which allows them to displace bacterial communities inhabiting concrete items (iii). Because of the chemical nature of citric acid, it has the ability to react with the alkaline components of the concrete matrix to form calcium citrate. The chemical reaction of citric acid with the top layer of calcium hydroxide creates the etching of the concrete surface. As long as there is excess acid present, the calcium citrate will remain soluble. As the acid is consumed, the solubility decreases and the calcium citrate precipitates out as a solid, inducing microcracks deepening. All mentioned factors cannot be compared to well-known destructive activity of sulfate-reducing bacteria in sewage, where the sulfuric acid formed from hydrogen sulfide dissolves the carbonates in the cured cement and causes strength loss, as well as producing sulfates which are harmful to concrete [20]. They also at least in part differ from variable biological factors influencing buildings in a sub-tropical climate [21]. Today, biofouling and biodeterioration of concrete structures including underground structures, sewage systems, at-sea structures, and wastewater treatment systems has been examined [5]. Great attention is paid to biodeterioration of painted buildings [17, 22] and objects of cultural heritage [21, 23]. Unfortunately, there is not enough information on biodeterioration of concrete items of residential buildings in temperate and continental climate. From this point of view, our work provides the first data characterizing destructive properties of fungi inhabiting concrete in these conditions.

### 4.3. Loss of Calcium Leads to Concrete Strength Decrease

Calcium release and acid production were known to be important processes influencing concrete stability. We observed the decrease of calcium content after* P. brevicompactum* P4 growth on the concrete, which was higher in case of growth in liquid phase compared to the superficial growth (Figure 5).* Fusarium* species induced the same alteration of concrete. Nonbiological formation of CaCO_3_ in the reaction of air-born carbon dioxide with the calcium hydroxide in concrete was shown on the concrete surface which was not infected by fungi. Stable CaCO_3_ was precipitated in the pore system (Figure 3(a)). Carbonatation has two effects: it increases mechanical strength of concrete, but it also decreases alkalinity, which creates favorable conditions for fungi growth leading to the loss of calcium in concrete (Figure 5). The 15 fungal isolates identified in this study produced oxalic acid (Figure 2(a)) and therefore form calcium oxalate which creates a protective layer on the concrete surface contributing to positive impact of this acid production [14, 24]. A lower number of isolates producing lactic and malic acids which form more or less soluble calcium salts indicates their prevalent role in pH decrease which promotes the future development of fungi. Citric acid can be attributed to agents of negative influence on concrete because calcium citrate precipitates increasing microcracks size. The main conclusion that could be made from the results concerning fungal acid production is the ambiguity of the effect of various acids on concrete stability. In respect that the acid spectra of different strains are different, it is difficult to predict structure damage without studying the characteristics of specific destructors. For example, the study of marine-grade concrete colonized with barnacle indicates that barnacles do not enhance but likely reduce rates of mechanical breakdown on rock and concrete by buffering near-surface thermal cycling and reducing salt ion ingress [25]. Bacterial biomineralization can contribute to protection-consolidation of ornamental stone [26]. Of course, fungi cannot be related to agents of bioprotection, but their influence on concrete is not always obligatory negative and depends on certain acid production.

Here, we have found that standard test recommended for assessment of concrete biodamage characterized the differential lesion of different concrete marks at the trend level. Nevertheless, the compressive and flexural strengths of concrete specimen М 1000 retain less alerted in comparison to other concrete brands (Table 1). If we focus on these data, the higher content of cement in the concrete sample increases its resistance to fungal harm. It was shown that the ability of concrete to resist damaging influence of hydrochloric acid is due to the cement brand and its amount [27]. Because microorganisms cause both the initiation, as well as the intensification of concrete corrosion processes [10], probably, the testing time was not enough to detect more significant differences in alteration of concrete strength characteristics.

### 4.4. Fungal Toxins Contribute to Sick Building Syndrome

Finally, it must be noted that the colonization of building material by toxigenic fungi raises the question of the subsequent exposure of occupants to aerosolized mycotoxins.* P. brevicompactum,* commonly encountered in the indoor air and isolated at out study, is known to produce mycotoxins. There are the mycophenolic acid [28] and ochratoxin A which has carcinogenic, teratogenic, and nephrotoxic potential and sustains a high half-life in human blood [29]. The most part of the aerosolized toxic load is found in particles whose size corresponds to spores or mycelium fragments. However, some toxins were also found on particles smaller than spores that are easily respirable and can deeply penetrate into human respiratory tract [30] contributing to sick building syndrome which is considered to be a multifactorial health problem of the overwhelming majority of the population in cities [31]. The relevance of the concrete biodeterioration study itself and its consequences for the environment and human health is constantly increasing.

## 5. Conclusion

Biodeterioration of concrete widely used in residential city buildings has now become an important environmental problem. First at all, fungal deterioration of houses increases during the operation time and is hard to stop. Second, its consequences for human health must be taken into account, as, according to statistics, urban residents spend about 95% of their time indoors. Here, we have characterized the metabolic activity of dominant fungal habitants of old city building contributed to concrete biodetrioration, including radial growth rate, antagonistic activity, and production of organic acids. We have demonstrated that fungal growth induced calcium release from concrete surface. Decrease of calcium was higher if the concrete surface was located in liquid phase compared (41%) to surface contacted with air (36%). The alterations of the compressive and flexural strengths of concrete specimens depending on concrete brands were detected along with decrease of calcium content. The stability of concrete samples exposed to growing* Aspergillus* and* Penicillium* isolates during 28 days decreased on about 1%.To detect real individual alterations of the concrete specimens induced by fungi, the duration of the recommended standard test has to be prolonged. The problem of concrete biodeterioration in city houses cannot be regarded as top-significant today, but the time factor enhances its value. Special emphasis on variable fungal destructive activities, on complex mechanisms, and on a system for correct analysis of concrete deterioration all are areas in need of additional investigation.

## Figures and Tables

**Table 1:** Alteration of strength characteristics of concrete during 28 days of exposure in Czapek Dox medium without inoculation (CDM) and inoculated by spores of *Aspergillus fumigatus* A2 and *Penicillium brevicompactum* P4.

Concrete specimens	Characteristics of concrete strengths, MPa							
	Compressive strength, R_c_				Bending strength, R_f_			
	Initial	CDM	A2	P4	Initial	CDM	A2	P4
М400	6.60	6.55	6.50	6.48	32.30	32.25	32.15	32.14
М500	7.10	7.05	7.00	7.00	42.80	42.75	42.65	42.60
М 600	9.50	9.45	9.40	9.40	60.80	60.72	60.70	60.69
М 800	10.50	10.49	10.40	10.42	74.80	74.80	74.70	74.65
М 1000	12.20	12.17	12.15	12.15	95.20	95.16	95.00	95.05

## Data Availability

The data used to support the findings of this study are available from the corresponding author upon request.
